# Clinicopathological Insights Into Endometrial Osseous Metaplasia: A Rare Case Report

**DOI:** 10.1155/crip/9296695

**Published:** 2025-08-17

**Authors:** Shifa F. Khan, Prachi R. Gaddam, Uma Chaturvedi, Raji T. Naidu, Susan Cherian

**Affiliations:** Department of Pathology, Bhabha Atomic Research Centre, Mumbai, Maharashtra, India

**Keywords:** case report, endometrium, histopathology, hysteroscopy, osseous metaplasia

## Abstract

Endometrial osseous metaplasia is a rare entity encountered in the reproductive age group characterized by the presence of mature bone within the endometrium. Most of the cases are associated with secondary infertility, with a past history of abortion or chronic endometritis. Various hypotheses, such as chronic inflammation, dystrophic calcification, and residual embryonic tissue, have been proposed for the etiopathogenesis. Hysteroscopic removal of the osseous tissue leads to the restoration of normal endometrial function and can potentially resolve infertility. We present a case of a 36-year-old female presenting with abdominal pain. Ultrasonography was suggestive of dystrophic calcification in the endometrium. Bony fragments, along with endometrial curettage material, were removed by hysteroscopy. Histopathology revealed proliferative endometrial glands and stroma admixed with fragments of mature bony trabeculae. A diagnosis of endometrial osseous metaplasia was confirmed. This case report highlights the importance of correctly diagnosing this rare condition on histopathology and differentiating it from other mimics to guide appropriate treatment.

## 1. Introduction

Endometrial osseous metaplasia is an uncommon condition characterized by the presence of mature bone in the endometrium [1]. It has an estimated incidence of 3/10,000, with only a handful of cases reported in the literature [2, 3]. Most of the patients are in the reproductive age group and present with complaints like abnormal uterine bleeding, dysmenorrhea, pelvic pain, vaginal discharge, or secondary infertility [4].

The pathogenesis of endometrial osseous metaplasia is not clear, but the most widely accepted hypothesis is metaplasia of the stromal cells into osteoblasts, which produce osseous tissue [2]. Cases can be managed by complete removal of bony spicules from the endometrial cavity with hysteroscopy [3]. Although rare, it is important that pathologists are familiar with this entity in order to diagnose it correctly.

## 2. Case Presentation

A 36-year-old woman presented with abdominal pain. She had an obstetric history of two full-term deliveries by caesarean sections and a medically terminated pregnancy followed by dilatation and curettage (D and C) at 8 weeks' gestation. Curettage was done 9 months prior to the present complaints. The ultrasonography report of the current visit was suggestive of dense dystrophic calcification in the subendometrial location in the midlower body region of the uterus. The urine pregnancy test was negative. General physical examination, routine hematological parameters, and serum calcium levels were within normal range. Diagnostic hysteroscopy showed a bone-like structure in the lower midbody region of the posterior wall of the uterus. Bony fragments, along with endometrial curettage material, were sent for histopathological examination.

**Figure 1:**
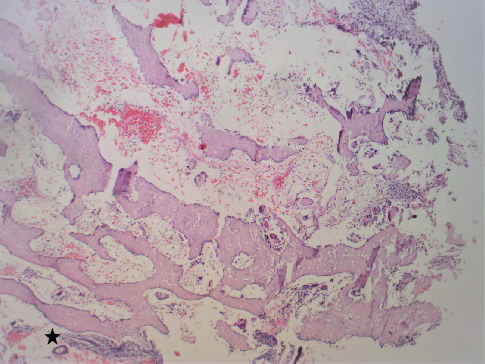
Photomicrograph showing bone trabeculae along with endometrium (marked with star) and hemorrhage (H and E, ×40).

**Figure 2:**
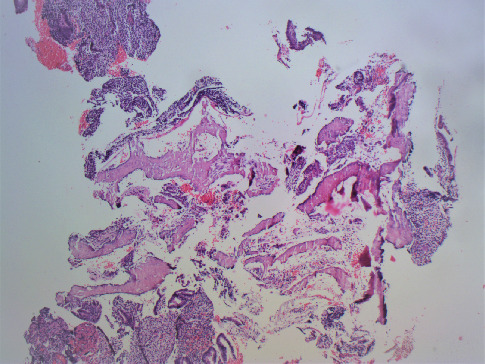
Photomicrograph showing osseous tissue intermixed with endometrial glands and stroma (H and E, ×40).

**Figure 3:**
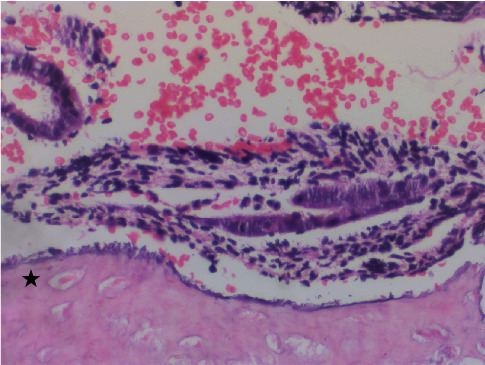
Photomicrograph showing proliferative endometrium with mature bone (marked with star) (H and E, ×400).

On gross examination, congested soft tissue bits, along with a few bony hard pieces aggregating to 1 × 1 × 1 cm, were seen. The tissue was processed directly without the need for decalcification. The hematoxylin and eosin–stained sections showed proliferative endometrial glands and stroma admixed with fragments of mature bony trabeculae (Figures 1 and 2). Additionally, there was hemorrhage, fibrin collection, and scattered inflammation (Figure 1). Necrosis, granuloma, products of conception, and cartilage were not seen. Features of malignancy, atypical glands, or hyperplasia were not seen (Figure 3). A diagnosis of proliferative endometrium with osseous metaplasia was given on histopathology.

The patient had an uneventful postoperative course and was relieved of abdominal pain on follow-up.

## 3. Discussion

Endometrial metaplasia is a nonneoplastic condition that can be categorized into epithelial and mesenchymal types. Mesenchymal metaplasia involves the formation of islands of mesenchymal tissue, such as smooth muscle, cartilage, bone, glial tissue, or adipose tissue, within the endometrial stroma [5]. Endometrial osseous metaplasia is a rare entity with unclear etiology and pathogenesis [3]. Most of the reported cases are in the reproductive age group, with more than 80% of cases presenting after pregnancy, while it is infrequently observed in postmenopausal women [6, 7]. Patients may present with a variety of symptoms, including infertility, menstrual irregularities, pelvic pain, and dyspareunia [8]. The presence of bony fragments in the endometrium can function as an intrauterine contraceptive device, leading to infertility. In most cases, the complete removal of heterotopic bony tissue from the uterus via hysteroscopy effectively restores fertility [9].

Several hypotheses have been suggested to explain the development of endometrial osseous metaplasia, including heterotopia, calcification from chronic inflammation or necrosis, ossification after postabortive endometritis, metastatic calcification, tissue repair–related metaplasia, extended estrogen exposure postabortion, and retained fetal bone fragments [10]. These theories can generally be divided into two main categories: the metaplastic transformation of endometrial stromal cells and the implantation of fetal tissue [11]. Chronic endometritis induces the release of superoxide radicals and tumor necrosis factor from inflammatory cells, which subsequently stimulate the proliferation of mesenchymal cells capable of undergoing metaplasia and differentiating into chondroblasts or osteoblasts [9]. Studies have confirmed that various cell types, including pluripotent mesenchymal cells, fibroblasts, and Müllerian cells, can undergo osseous metaplasia in response to inflammation and curettage. A DNA analysis conducted by Cayuela et al., comparing bone fragments from osseous metaplasia lesions and surrounding endometrium, found that the metaplastic bone is of maternal origin—thereby challenging the theory of implantation of fetal tissue [12]. Additionally, most case reports have found no association with hypercalcemia or related metabolic disorders, which undermines the metastatic calcification hypothesis [4, 10]. These findings strongly support the hypothesis of metaplastic transformation of endometrial stromal cells.

Histopathological examination shows mature or immature bone tissue surrounded by normal endometrium [3]. Metaplastic, nonneoplastic lamellar or trabecular bone is characterized by endogenous bone development, which may also show extramedullary hemopoiesis [1, 2]. The endometrium within the bone tissue responds well to cyclical stimulation in the presence of the heterotopic tissue [9]. Minimal or no tissue reaction is seen in the biopsies reported in the literature, as well as in the present case [2, 10]. The absence of endochondral ossification or primitive fetal tissues, with minimal inflammatory reaction, is helpful to differentiate osseous metaplasia from retained fetal tissue.

Dystrophic calcification due to endometrial tuberculosis is an important differential diagnosis, especially in endemic regions like India. Abundant chronic inflammatory granulomatous infiltrates, giant cells, and interspersed foci of calcification are characteristic of endometrial tuberculosis. Detection of acid-fast bacillus-specific nucleic acid in endometrial tissue is confirmatory for endometrial tuberculosis. Calcific endometritis, due to dystrophic calcification of retained necrotic tissue, is a distinct histopathological entity and needs to be distinguished from osseous metaplasia of the endometrium [13]. The present case did not show fetal parts or granuloma on histology.

The absence of a mass lesion on ultrasonography is helpful for pathologists to exclude the diagnosis of uterine teratomas, mixed mesodermal tumors, calcification within leiomyoma, and malignant mixed Müllerian tumors on a biopsy specimen. Recognizing the nonneoplastic nature of this condition is crucial to prevent misdiagnosis as a heterologous sarcomatous component of uterine carcinosarcoma [5].

Ultrasonography plays an important role in the diagnosis of osseous metaplasia due to its availability, wider acceptance, and being a noninvasive procedure [2, 14]. The distinct hyperechogenic pattern with posterior acoustic shadowing suggests the presence of osseous tissue in the uterus [2]. Recommended treatment involves the complete removal of the bony spicules through hysteroscopy, including any fragments that may be embedded in the myometrium [10]. Histopathology is essential to establish the final diagnosis and to differentiate it from other conditions, as discussed above.

## 4. Conclusion

Osseous metaplasia of the endometrium is an uncommon condition that can be successfully managed with hysteroscopic resection, providing a potential for fertility restoration in young patients. Diagnosis can be suggested by ultrasonography and confirmed by histopathology. It is essential for surgical pathologists to be aware of this nonneoplastic condition to differentiate it from other inflammatory conditions and avoid misdiagnosis of malignancies like carcinosarcoma. This report underscores the importance of correctly diagnosing osseous metaplasia on histopathology with correlation of ultrasonography and hysteroscopy findings to guide appropriate patient management.

## Data Availability

Data sharing is not applicable to this article as no datasets were generated or analyzed during the current study.
